# Prevalence and Socio-Demographic Predictors of Food Insecurity in Australia during the COVID-19 Pandemic

**DOI:** 10.3390/nu12092682

**Published:** 2020-09-02

**Authors:** Katherine Kent, Sandra Murray, Beth Penrose, Stuart Auckland, Denis Visentin, Stephanie Godrich, Elizabeth Lester

**Affiliations:** 1 Centre for Rural Health, University of Tasmania, Tasmania 7250, Australia; stuart.auckland@utas.edu.au; 2 School of Health Sciences, University of Tasmania, Tasmania 7250, Australia; Sandra.murray@utas.edu.au (S.M.); denis.visentin@utas.edu.au (D.V.); 3 Tasmanian Institute of Agriculture, University of Tasmania, Tasmania 7000, Australia; beth.penrose@utas.edu.au; 4 School of Medical and Health Sciences, Edith Cowan University, Western Australia 6230, Australia; s.godrich@ecu.edu.au; 5 Institute for Social Change, University of Tasmania, Tasmania 7000, Australia; elizabeth.lester@utas.edu.au

**Keywords:** food insecurity, Australia, COVID-19, food supply

## Abstract

The COVID-19 pandemic has exacerbated economic vulnerabilities and disrupted the Australian food supply, with potential implications for food insecurity. This study aims to describe the prevalence and socio-demographic associations of food insecurity in Tasmania, Australia, during the COVID-19 pandemic. A cross-sectional survey (deployed late May to early June 2020) incorporated the U.S. Household Food Security Survey Module: Six-Item Short Form, and fifteen demographic and COVID-related income questions. Survey data (*n* = 1170) were analyzed using univariate and multivariate binary logistic regression. The prevalence of food insecurity was 26%. The adjusted odds of food insecurity were higher among respondents with a disability, from a rural area, and living with dependents. Increasing age, a university education, and income above $80,000/year were protective against food insecurity. Food insecurity more than doubled with a loss of household income above 25% (Adjusted Odds Ratio (AOR): 2.02; 95% CI: 1.11, 3.71; *p* = 0.022), and the odds further increased with loss of income above 75% (AOR: 7.14; 95% CI: 2.01, 24.83; *p* = 0.002). Our results suggest that the prevalence of food insecurity may have increased during the COVID-19 pandemic, particularly among economically vulnerable households and people who lost income. Policies that support disadvantaged households and ensure adequate employment opportunities are important to support Australians throughout and post the COVID-19 pandemic.

## 1. Introduction

Food security is achieved “when all people, at all times, have physical, social and economic access to sufficient, safe and nutritious food that meets their dietary needs and food preferences for an active and healthy life” [1]. This broad definition emphasizes four distinct dimensions of food security [2] which include the availability, accessibility and utilization of food, in addition to the stability of each of these factors, which refers to an ability to withstand shocks to the broader food system. Food insecurity occurs when at least one of these domains are not met, where the experience at a household level may be temporary or longer-term [3]. Access to adequate food is a core social determinant of health, and food insecurity is related to poor nutritional intake and higher mortality rates [4,5]. Even temporary reductions in food security can impact long term health and cause loss of human capital, which can take years to recover from [6].

Australia is a developed country with proficiency in food production, but despite this, it had been conservatively estimated that between 4–5% [7,8] of the Australian population experienced food insecurity prior to the COVID-19 pandemic. Higher proportions have been reported using more accurate measurement tools in many different sub-populations across Australia [9]. Both in Australia and internationally, independent household-level determinants of food insecurity include low income [10] and sudden reductions in income [11], in addition to other demographic factors, such as household structure and education [12]. Increased levels of food insecurity have been documented during periods of economic downturn, even after controlling for comprehensive socio-demographic factors [13].

The COVID-19 pandemic has been a catalyst for unprecedented social and economic change across the world, which may exacerbate levels of food insecurity in the short- and longer-term. Australian governments responded with wide-spread public health measures, including travel-bans, lockdowns, school closures, and other social distancing restrictions [14], which have minimized the rate of COVID-19 transmission in comparison to other countries in the first six months of the pandemic. However, in addition to global economic factors, these measures also triggered a sudden and significant loss of employment, underemployment, and income reductions for a considerable proportion of the population. Between 14 March and 27 June 2020, the number of payroll jobs reduced by 5.7% Australia-wide, which indicates an unprecedented decrease in the number of people receiving wages [15]. Consequently, Australian households have experienced heightened economic vulnerability and it has been reported that half of Australians (49%) are drawing on unsustainable, finite resources (such as credit or superannuation) to manage their household expenses, which is likely to reduce the amount of money available to be spent on food [16]. In response, the Australian government introduced relief measures to ameliorate the loss of income, including JobKeeper (a payment to businesses to maintain employment) and the Coronavirus Supplement (an increase in government support payments including for those unemployed—called Jobseeker). The Australian food supply was also temporarily disrupted at the beginning of the COVID-19 pandemic due to changing consumer purchasing patterns and stockpiling of food [17], which reduced the availability of food. Access to food outlets was restricted as many hospitality businesses were forced to close, due to social distancing restrictions [18]. The capacity to shop for food for other individuals (e.g., the elderly and immunocompromised) was reduced in an attempt to avoid the risk of infection. Collectively, these factors have the potential to worsen the overall prevalence of food insecurity and may exacerbate or change the socio-demographic predictors associated with food insecurity in Australia. In the longer-term, the impact of the economic slowdown as a result of changed employment and reduced consumer spending throughout the COVID-19 crisis, in addition to challenges related to food production and supply chains may further exacerbate the experience of food insecurity both in Australia and other countries.

As food insecurity has adverse effects on health [19], measurement of its prevalence during the COVID-19 pandemic, including the levels of severity, is important. Accurately understanding the magnitude of the issue and its determinants, can inform the development of targeted and effective strategies to minimize food insecurity in Australia. Therefore, a cross-sectional study was conducted in Tasmania, Australia, to determine the prevalence and socio-demographic predictors of food insecurity during the COVID-19 pandemic.

## 2. Materials and Methods

### 2.1. Study Setting

Tasmania is an island state of Australia, located below the south-eastern coast of the mainland, with a population of just over 500,000 residents [20]. In Tasmania, the population is 51% female and 49% male, and the median age is 42 years, which is older than the Australian median age of 38 years. The pre-COVID-19 unemployment rate was approximately 7%, and 18.8% of the Tasmanian population have their occupation listed as “professionals” [20]. The median household weekly income in 2016 was AU$1,100, which is lower than the Australian median household weekly income of AU$1,438 [20]. The predominant household composition is coupled families without children (43%) and coupled families with children (38%) [20].

### 2.2. Data Collection

This study was conducted in collaboration with The Tasmania Project, a University of Tasmania initiative established by the Institute for Social Change to understand how Tasmanians are experiencing and adjusting to the social, political, and economic responses to COVID-19. A cross-sectional survey was conducted with a non-random sample of adult Tasmanian residents (aged 18 years and over) between 25 May and 7 June 2020. Convenience sampling methods were used to recruit participants, with the survey link being promoted through social media and disseminated through community groups. In addition, an email invitation was distributed to potential participants who had signed up for updates related to The Tasmania Project. Lastly, interviews with traditional media outlets were used to promote the survey and research to the wider Tasmanian community.

Participants used a link to enter the survey and were provided with a participant information sheet. Participants were then asked, “Have you read the information provided in the Participant Information Sheet and do you freely agree to participate in this project?”. Participants who selected “no” could not proceed. Participants were also screened for eligibility to ensure they were aged 18 years and over and were currently living in Tasmania, Australia. Eligible, consenting participants completed an online, self-administered survey through SurveyMonkey.

### 2.3. Measures

The survey used the U.S. Household Food Security Survey Module: Six-Item Short Form (HFSSM) to determine the prevalence of food insecurity [21]. This validated screening tool [22] sought responses to six questions self-reporting of uncertain, insufficient or inadequate food access, availability and utilization, due to limited financial resources, and the compromised food consumption that may result. The HFSSM generally applies a reference timeframe of 12 months; however, it was less applicable for this study which was conducted at the beginning of the COVID-19 pandemic. Therefore, the acceptable shorter reference period of 30 days was selected to capture the prevalence of food insecurity during the COVID-19 pandemic only [23]. Responses to the six questions were coded and assessed in accordance with the user notes [23], where each affirmative response was assigned a score of 1, and summed raw scores were used to describe food insecurity at the household level. Scores were then used to categorize respondents as having high (0), marginal (1), low (2–4) or very low food security (5–6). In previous Australian studies [24], scores of 0 and 1 have been grouped together, while international studies have reported these scores separately. Our analyses kept these scores separate, due to the short reference period, and the fact that the majority of respondents who scored 1 reported running out of food and could not afford to buy more. An affirmative response to this question would indicate household food insecurity with other scales, including the single-item measure of food insecurity which is commonly used in Australian studies of food insecurity [9].

Fifteen socio-demographic variables were collected, including age (in years), gender (male, female, other), local government area of residence, if they identified as from Aboriginal and/or Torres Strait Islander descent, whether they have a health condition or disability that limited their activity, citizenship status (born in Australia, Australian citizen, permanent resident or temporary resident), the highest level of education, relationship status, household composition, employment status, total household income bracket and whether they were the main shopper for their family. Additional questions asked respondents about how the COVID-19 pandemic affected their household finances and job status, including whether the COVID-19 pandemic resulted in a change in their employment and/or a decrease in household income, and whether they were receiving COVID-19 related Australian government benefits (JobKeeper or JobSeeker). The study was conducted in accordance with the Declaration of Helsinki, and the protocol was approved by the University of Tasmania’s Social Sciences Human Research Ethics Committee (Ethics Project ID: 20587).

### 2.4. Data Analysis

Data sets were exported from the online survey platforms to IBM SPSS Statistics for Windows, version 26.0 (IBM Corp., Armonk, NY, USA), cleaned and prepared for statistical analysis which was performed in Stata 14.2 (Statacorp, 2015). All available survey data were used in the analyses. The significance level for all analyses was set at *p* < 0.05.

**Figure 1:**
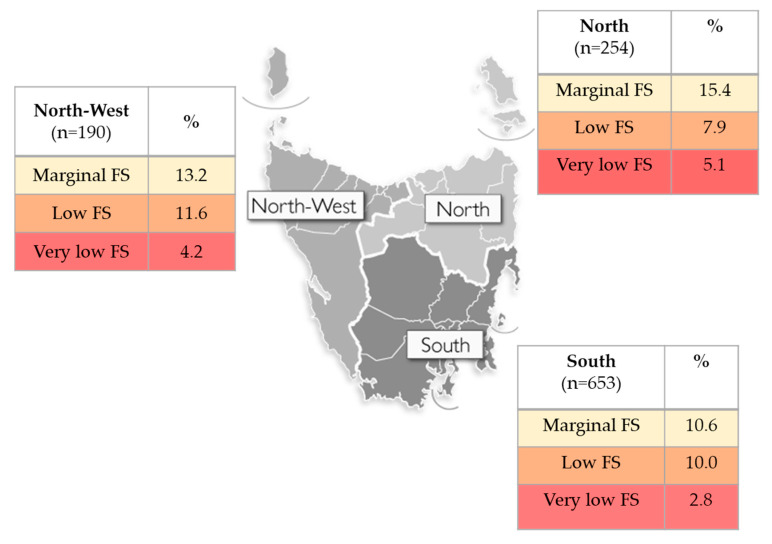
Prevalence of Marginal, Low and Very Low Security by region in Tasmania, Australia (map sourced from Department of Health and Human Services Tasmania: http://www.dhhs.tas.gov.au/tho). FS—Food Security.

A binary variable of food security status was generated for application in the univariate and multivariate logistic regression, where food secure was determined by a score of 0, and food insecure was a score of 1–6 on the HFSSM. In addition, several of the socio-demographic variables were recoded into a smaller number of categories, due to low cell counts and for ease of interpretation. Recoded variables included coding thirty Local Government Areas (LGA) of residence categories into three regions (North, South, North-West and West) (see Figure 1 for graphical representation) and rurality (urban and rural dwelling regions). Age categories were developed from the continuous variable (18–25 years, 26–35 years, 36–45 years, 46–55 years, 56–65 years, 65+ years). Disability status was recoded from three options (no, yes a little, yes a lot) to two (yes, no) by collapsing the affirmative responses. Relationship status was recoded from seven to four groups (married/de facto, never married, previously married, living apart) by combining widowed, divorced, and separated into previously married. Household composition was reduced from ten options to five (couple with no dependents, couple with dependents, single adult with dependents, single person house, other (group/share)). Highest education status achieved was recoded from eight options into three (university degree, diploma/Technical and Further Education (TAFE) qualification, high school qualification). Employment status was recoded from eight options to three (employed [including self-employed]), unemployed and other [which included student, volunteer, retired and other]). Household income was recoded into three categories (AU$ <40,000, 40,000–80,0000 and 80,000+).

All socio-demographic variables were either categorical or ordinal and were cross-tabulated and summarized with frequencies and proportions. Cross-tabulations with Chi-square statistic were employed to generate descriptive statistics related to food security status, including responses to each of the six food security questions, and with each of the socio-demographic variables. Univariate logistic regression was performed individually for each socio-demographic characteristic to generate unadjusted odds ratios for food insecurity. Correlation coefficients between all variables were calculated to demonstrate the interrelationships and inform the multivariate analysis. A multivariable logistic regression was performed, including all measured variables to yield adjusted odds ratios (AOR) for food insecurity. Variables were retained in the final model if any level of the variable had *p* < 0.1.

## 3. Results

### 3.1. Sample Demographics by Food Security Status

Almost three-quarters of the respondents (*n* = 1170) were categorized as having high security (*n* = 863, 73.9%), with 12.3% reporting marginal food security (*n* = 144), 10.1% low food security (*n* = 118) and 3.7% (*n* = 43) having very low food security. Socio-demographic characteristics of the survey respondents according to food security categories and assessments of the impact of the COVID-19 pandemic on employment and income are presented in Table 1. The majority of respondents were female (77%), and a large proportion (68%) were aged over 46 years. Most respondents (69%) were in a married or de facto relationship, and 28% of respondents had dependents living with them. With regards to income, 31% of respondents had incomes over AU$100,000 per year, and a majority (67%) had a university education (Bachelor’s degree or higher). Main household shoppers were the predominant survey respondents (82%).

The proportion of respondents in food secure groups rose with increasing age (Table 1). A significantly greater proportion of younger respondents aged 18–25 years were classified as having low (29%) or very low food security (7%) over the past month in comparison to older age brackets. While there was no significant difference between men and women in the food secure categories (~74%), a greater proportion of female respondents reported very low food security (4%) in comparison to males (1%). A significantly greater proportion of respondents with a disability reported experiencing low and very low food security (14% and 10%, respectively) in comparison to respondents without a disability (9% and 2%). A small proportion of respondents identified as Aboriginal and/or Torres Strait Islander (2.3%), but less than half of these respondents were food secure, and more than 1 in 5 of these respondents reported experiencing low and very low food security, which was statistically significantly greater than those who did not identify as Aboriginal and/or Torres Strait Islander (Table 1).

A significantly greater proportion of university-educated respondents had high food security (80%) in comparison to smaller proportions of those with a diploma/TAFE qualification (64%) and those who obtained a high school qualification (61%). A significantly larger proportion of Australian citizens born in Australia (74%) and overseas (81%) were classified as having high food security in comparison to permanent residents (68%) and temporary residents (41%). Of note, are the high proportions of temporary residents in the marginal (18%), low (24%) and very low (18%) food security groups in comparison to other residents. Significantly higher proportions of those respondents who were never married (9%) and separated (15%) were in the very low food security group, and only 54% of respondents in households of single adults with dependents were classified as having high food security. Half (50%) of respondents on the lowest income were classified as having high food security, with a further 21% in the low food security group and 16% in the very low food security group, which is much higher than other income brackets (Table 1).

Similar proportions of respondents who indicated that the COVID-19 pandemic had impacted their job were classified as having marginal food security (13%) in comparison to respondents without a job change. However, a significantly higher proportion of those whose job had been impacted by the pandemic were in the low (17%) and very low (5%) food security groups. Fewer respondents who had lost income as a result of the COVID-19 pandemic (34%) had high food security Table 1). Of note, 65% of respondents who had lost over 75% of their income reported some degree of food insecurity. Interestingly, a similar proportion of respondents receiving the JobKeeper government benefit had high food security, in comparison to less than half (48%) of respondents receiving the lower JobSeeker payment (48%), with 25% of these respondents classified as having marginal food security and a further 27% in the low and very low food security groups.

The prevalence of household food insecurity ranged from 23% in the South, to 29% in the North-West and West regions (Figure 1). The greatest proportion of respondents with very low food security were in the North (5%), compared with 4% in the North-West and West and 3% in the South. Despite a lack of statistically significant regional differences, a larger proportion of respondents residing in rural regions (Table 1) were classified in the marginal, low and very low food security groups (33%) in comparison to their urban-dwelling counterparts (23%).

The proportions of respondents in each food security group who provided affirmative responses to each of the six HFSSM questions are represented in Table 2. Of the six questions, marginally food secure respondents were most likely to respond affirmatively to the first question: “The food that (I/we) bought just didn’t last, and (I/we) didn’t have money to get more”. However, none of the respondents in the marginally food secure group reported eating less than they wanted or being hungry because there was not enough money for food. In comparison, nearly all respondents (95%) in the very low food security category responded affirmatively to the first question, and a further 75% reported experiencing hunger. In addition, 90% of respondents in the low food security category reported they cut down on food or skipped meals for least three days in the past 30 days because they could not afford to buy more, but only 10% in this group reported experiencing hunger. While the majority of respondents in the low food security group (81%) did not cut down on the size of meals, 94% reported being unable to afford to eat balanced meals.

### 3.2. Socio-Demographic Predictors of Food Insecurity

Table 3 presents crude and adjusted odds ratios of household food insecurity for the variables considered. Adjustment for socio-demographic characteristics considered in our multivariable model yielded modest decreases in the strength of some effects, highlighting the co-occurrence of factors associated with elevated risk, especially for income variables. In the multivariate analysis, two demographic variables were removed from the final model, which were also not independently significantly associated with higher food insecurity (main shopper, region). The region was also highly colinear with rurality, which was retained (see Table 4 for correlations between variables). As the COVID variables were assessing different aspects of the effect of COVID-19 on income, only one (income decrease) was retained in the final model, due to collinearity. The adjusted model had Pseudo R^2^ = 0.152, and Likelihood ratio test statistics χ^2^ = 159.6, *p* < 0.0001.

The adjusted model indicates that increasing age was protective against food insecurity, with the odds of experiencing food insecurity reducing by 16% with every decade of life. Respondents who identified as Aboriginal and/or Torres Strait Islander had more than three-fold greater odds of experiencing food insecurity; however, after adjustment for other household characteristics, this difference did not remain significant despite the AOR of 2.4, which may reflect the small proportion of respondents who identified as Aboriginal and/or Torres Strait Islander. Respondents with a disability had more than two-fold increased odds of experiencing food insecurity compared with those without a disability, which was modestly reduced (AOR: 1.7) after adjusting for other characteristics. An 82% increase in the odds of experiencing food insecurity was evident for respondents in rural areas after adjusting for other characteristics. Higher levels of education were protective against food insecurity, with respondents with a diploma/TAFE or high-school qualification showing a two-fold increase in the odds of experiencing food insecurity compared to those with a university-level education (Bachelor’s degree or higher), which remained significant after adjusting for other characteristics.

Temporary residents had a four-fold increase in the odds of experiencing food insecurity compared to Australian citizens, which was modestly reduced after adjusting for other characteristics and did not remain significant. The multivariate model showed that respondents who were previously married had two-fold higher odds of food insecurity than respondents who were currently married or in a de facto relationship. Compared to couple families without dependents, all other household types had increased odds of food insecurity. Interestingly, the odds ratio for food insecurity associated with being a single parent household fell from 3.22 to 1.17 with adjustment for other household characteristics, and this group was no longer statistically significantly different from other household types.

Household income was independently associated with food insecurity, with incomes above AU$80,000/year seemingly protective against food insecurity, and income below AU$40,000 per year associated with a two-fold increase in the odds of food insecurity. Respondents who reported that the COVID-19 pandemic had resulted in a change in their employment had a 75% increase in the odds of experiencing food insecurity in comparison to those not impacted. In comparison to those who were employed and not receiving government benefits, respondents who were receiving the JobKeeper government support payment had 20% higher odds of experiencing food insecurity, and those who were receiving Jobseeker support payments (a smaller fiscal amount) had a three and a half-fold increase in the odds of experiencing food insecurity. A gradient was apparent for respondents who had lost income as a result of the COVID-19 pandemic, where an income loss of 25% or more significantly increased the odds of experiencing food insecurity. Independently of other factors, including household income, a loss of more than 75% of income was associated with a seven-fold increase in the risk of food insecurity. In the adjusted model, similar effects for income decrease were observed, demonstrating that income loss is independently associated with higher odds of food insecurity.

## 4. Discussion

This study presents results from a survey of adults in Tasmania, Australia, assessing the prevalence and socio-demographic predictors of household food insecurity during the COVID-19 pandemic. Our results demonstrate that between late April and early June 2020, a time when wide-spread social distancing restrictions were in place, more than 1 in 4 (26%) respondents had experienced food insecurity to some degree. Concerningly, 14% of respondents experienced more severe food insecurity, which meant they were regularly going hungry and were unable to afford balanced meals over the previous month. These statistics are substantially higher than the 2019 Tasmanian food insecurity prevalence of 6.2%, preceding the COVID-19 pandemic [25], and higher than the national reported prevalence of 4% [7]. Comparable to the results of our study, previous research has shown that food insecurity was highest in the North (6.9%) of Tasmania, and lower in the South (6.1%) and North-West (5.8%) [25]. The 2019 Tasmanian survey used a single item food insecurity question, meaning these results are not directly comparable to the current study. However, given that more than 20% of respondents to our survey provided an affirmative response to the equivalent question (see Table 2), this indicates that the burden of food insecurity in Tasmania is substantially higher than pre-COVID-19 levels.

There are emerging reports of much higher levels of food insecurity being experienced by populations across the world during the COVID-19 pandemic. A cross-sectional study of low-income adults in the US reported that 20% of respondents were marginally food insecure, and a further 44% were food insecure [26]. However, this study was limited by its focus on low-income households, and the results are, therefore, not generalizable to the wider population. Our results demonstrate that the experience of food insecurity was not limited to only those on low incomes, and that loss of income at any level above 25% contributed to substantially higher odds of experiencing food insecurity. Results from a survey in the north-eastern US state of Vermont, identified a 33% increase in food insecurity since the beginning of the pandemic, with 24% of households experiencing food insecurity (up from 18%) [27]. A Canadian survey [28] conducted during April–May 2020 found that almost one in seven (14.6%) Canadians experienced food insecurity to some degree in the previous 30 days, and those who had reduced employment due to COVID-19, were more likely to be food insecure (28.4%) than those who were working (10.7%) [28]. A study in the UK reported that 16.2% of adults had experienced food insecurity since the COVID-19 lockdown began [29], up from 7.6% in 2018. Interestingly, in this study, a lack of food available in the shops contributed to 40% of food insecurity experienced, highlighting the importance of stability in the food supply as an overarching domain of food security. The proportions of more severe food insecurity were comparable with our study, where it was reported that 10% of adults skipped meals, and 4% regularly went without food [29]. Our study was unable to determine the extent to which food shortages, resulting from food hoarding, impacted food insecure responses. However, it is likely that food-insecure households were left at an extreme disadvantage because of food hoarding. In addition to the wide-spread unavailability of some foods, Australian retail outlets and supermarkets placed strict limits on the amount of staple food items that could be purchased in one transaction, which further reduced the ability of households to buy adequate food, especially for larger families living far away from their nearest shop, who need to buy in bulk. While an investigation of the coping strategies adopted by food-insecure households was not a focus of our study, the fact that many respondents reported running out of food, but did not report being hungry shows coping strategies being employed to some degree, especially for those in the marginal and low food security groups (Table 2). Such strategies could have included accessing emergency food relief, in addition to restaurants and community groups providing no-cost or low-cost meals and food boxes.

Our analyses demonstrate that the higher probability of household food insecurity was closely associated with many socio-demographic factors, especially financial factors specific to the COVID-19 pandemic. The most substantial factor in our regression analyses was loss of income related to COVID-19, with respondents who had lost the majority of their income showing up to a seven-fold increase in the odds of food insecurity. Similarly, in the UK, adults reported that loss of income above 50% resulting from the COVID-19 pandemic were found to be at significantly higher risk of food insecurity, even after accounting for background socio-economic status [29]. Prior to the COVID-19 pandemic, 12% of Tasmanian households were reportedly in financial stress, which means they did not have or could not raise AU$2000 in an emergency [25], with this factor closely related to food insecurity in the state. Food insecurity has worsened within economically vulnerable populations under COVID-19 conditions, with a loss of income in already low-income households putting individuals at even higher risk of food insecurity. Additionally, COVID-19 has also created new economic vulnerability for people were previously food secure and who are now experiencing income losses. Previous research has shown that loss of an income or large household bills (also known as bill shock), can require temporary reallocation of financial resources away from groceries, which can result in food insecurity [10]. Previous research during global recessions has shown increased household food insecurity, which can take years to return to pre-recession levels [30]. Our study is limited in that we were unable to control for household savings or other assets, which may explain a large variation in a household’s ability to adjust to periods of economic shock in comparison to others. However, low-income households are unlikely to ever accrue substantial savings or assets which could cushion against financial shocks. Our analyses demonstrate that a substantial proportion (35%) of our respondents’ employment status had been impacted by the COVID-19 pandemic, and that these respondents were at higher risk of moderate to severe food insecurity. Positively, government financial support payments were being received by 14% of our respondents and those receiving the higher JobKeeper payment through their employer showed a similar level of food insecurity to those currently employed. However, approximately half of respondents receiving the lower JobSeeker payments reported experiencing food insecurity. This finding is somewhat at odds with national reports of how this new government supplement, which is higher than the usual unemployment benefits, has reduced financial and personal distress, and reduced food insecurity for people who normally access unemployment schemes [31]. However, the majority of respondents to the national survey had been receiving an income support payment for more than two years, and therefore, our results may reflect the experience of individuals newly claiming this scheme [31].

In published research, low income is the most consistent and often the strongest predictor of food insecurity [32]. In our study, those who were food insecure are more highly represented among lower-income brackets, especially for those on incomes lower than AU$40,000 per year. Incomes above AU$80,000 per year, which is higher than the median household income in Tasmania of approximately AU$57,000 [20], were associated with lower odds of food insecurity. Low-income households may be larger, and therefore, may be unable to purchase sufficient food to meet their needs, or they may be required to purchase smaller quantities of food, which are sold at a higher unit price, further increasing the prevalence of food insecurity in these households. Interestingly, food insecurity was still evident in respondents with the highest incomes (>AU$100,000 per year), indicating that income does not always reflect the economic conditions of the household. Of note, the increased food insecurity reported in our study and other studies during the COVID-19 pandemic suggests that the financial impact of COVID-19 on food insecurity is far-reaching in the population, and has affected both households with high socio-economic risk of food insecurity and those not typically perceived to be at risk of food insecurity. In addition, our analyses demonstrate that younger age, rurality, disability, lower education levels, and having dependents were also all independently associated with food insecurity, indicating that the socio-demographic and COVID-related factors cannot be explained merely in terms of their association with income. Other factors which were associated in the univariate analysis, but not the multivariate, included being of Aboriginal/Torres Strait Islander decent, temporary residency, and being single (never married or previously married). For these factors, the relationship with food insecurity may be due to lower-incomes and other factors.

In our study, increasing age was protective against food insecurity. These results align with published literature, which demonstrates that even after controlling for economic factors, the probability of moderate or severe food insecurity decreased with age [8,33]. Studies of older Australians have similarly reported a lower prevalence than in the general population, with prevalence rates of 3% for Australians aged over 65 years [34] and 2% of Australians aged over 65 years [35]. Compounding this effect, the impact of the COVID-19 pandemic has disproportionately affected younger Australians [16], due to a large reduction in casual and part-time jobs predominantly held by younger people (e.g., hospitality). Interestingly, our study showed that gender was not associated with an increased risk of food insecurity. This is somewhat at odds with the results of other studies, which have explained the increased burden of food insecurity experienced by women to be associated with gender-related economic factors, including lower employment opportunities and child-related duties [36]. Additionally, higher educational attainment was independently associated with lower odds of experiencing food insecurity, which is consistent with other Australian research [8,12], and international studies of food insecurity during the COVID-19 pandemic [26]. In line with our findings, the greater likelihood of food insecurity among Australians from Aboriginal and/or Torres Strait Islander descent has been documented previously [37], with the prevalence of food insecurity ranging from 76% in remote areas [38] to 20% in the state of Victoria [39]. Additionally, respondents reporting health conditions and disabilities that limited their daily activities were more likely to be food insecure than those without a disability and were also shown to experience more severe forms of food insecurity. These results are echoed in previous Australian [40] and international studies [41] from before the COVID-19 pandemic. While our study did not examine the cause of food insecurity in this group, an UK survey during COVID-19 identified that respondents with a disability had between two and four-fold increased risk of experiencing food insecurity as a result of economic hardship, a lack of food available in shops, and social isolation [29]. Respondents living in rural areas experienced a higher burden of food insecurity in comparison to respondents living in urban areas and were 80% more likely to be food insecure after accounting for other socio-demographic factors. In rural Australia, fresh and healthy food is very expensive in some areas, due to transportation and storage costs [42]. During the COVID-19 pandemic, reduced access to food and fewer shops in these areas was coupled with media reports of price gouging of foods in response to increased demand [43], and these factors may have infringed upon the ability of rural residents to buy enough healthy food to meet their needs.

Pre-COVID-19 evidence has inconsistently linked immigration status to food insecurity [8,44]. In our study, temporary residents were four times more likely to experience food insecurity compared with Australian citizens, which did not remain significant after controlling for other variables. This may either reflect the small number of temporary residents who responded to our survey, or reflect the exacerbated economic hardship experienced by temporary residents during the COVID-19 pandemic. In Australia, temporary residents were ineligible for government support payments, and were therefore, at a much higher risk of food insecurity due to loss of income. Comparable with published research [8,30], being married or in a de facto relationship was negatively associated with food insecurity. During the COVID-19 pandemic, this may be related to the increased financial buffer of two potential income streams in comparison to those in single adult households, or that our study investigated household income, not income per-person, so the adjustment for income may be less appropriate for single respondents. Additionally, we found that households with dependents were more likely to be food insecure compared with those without dependents. Interestingly, households headed by a single parent were at three-fold greater risk of food insecurity. However, after adjustment for other variables, this difference was no longer significant, indicating that other variables, such as lower-income or loss of income accounted for this increased risk during the COVID-19 pandemic.

This study has a number of strengths. To our knowledge, this may be one of the few studies that have examined the impact of the COVID-19 pandemic on food insecurity in Australia. Moreover, we were able to assess the prevalence of food insecurity in a large sample size relative to the population of Tasmania, using a multiple item food security tool, which has shown to be more comprehensive than the single item food security question applied in many Australian studies [24,45]. Despite these strengths, these findings must be considered within the context of a number of potential limitations. Our study was cross-sectional in nature, and therefore, our analyses are purely descriptive, and inferences are limited by the design of the study [46]. Importantly, despite the wide recruitment methods used, the likelihood of participating in the survey may be associated with food insecurity (participation bias), and the use of an online survey may have excluded some groups, including those with low literacy or people without internet access. Further, our sample may not be representative of the wider Tasmanian population [20], as our sample contained a higher proportion of female respondents (76.7%) compared with the demographic profile of the Tasmanian population (51.1% female). This over-representation may be explained by the food-based theme of the survey and that women have been reported to predominantly manage household meals [36]. Despite this, our results are supported by reports that women provide more reliable estimates of the food insecurity experiences of a household [47]. Our sample shared similar proportions of married and separated respondents with the Tasmanian population (46.0% married, 20.3% previously married, and 33.7% never married) [20]. However, our survey had a lower proportion of respondents who identified as Aboriginal and/or Torres Strait Islander (2.3%) than the wider Tasmanian population (4.6%), and a lower proportion of unemployed respondents (5.1%), compared to the general Tasmanian population (7%). Our respondents were overall very highly educated, with 67% having a university education, compared with 16.2% having tertiary qualifications in the wider Tasmanian community [20], which may be a result of our convenience sampling methods. As higher education has shown to be protective against food insecurity, actual levels of food insecurity may be higher in the general Tasmanian population. Lastly, the 6-item HFSSM is slightly less reliable than 18-item measure, does not measure the most severe levels of food insecurity and does not measure the food security of children in the household [21].

### Where to Next?

As the COVID-19 pandemic continues around the world, it is likely that more severe economic vulnerabilities will emerge towards the end of 2020 and beyond. Further monitoring of food insecurity across Australia and internationally is needed to support the ongoing recovery from the COVID-19 pandemic. Prior to the pandemic, food-insecure households have reported numerous coping strategies for making ends meet, including seeking resources from within their social network in addition to emergency food relief. However, social distancing restrictions, business closures and other public health measures may infringe upon these coping strategies, and newly food-insecure households may not have appropriate knowledge of support services. Therefore, further Australian research is urgently needed to examine the coping strategies that food-insecure households are utilizing during the COVID-19 pandemic, including the appropriateness of income support payments for alleviating more severe food insecurity during the COVID-19 pandemic.

Our results indicate higher levels of food insecurity in Australia during the COVID-19 pandemic and could inform responsive policy interventions. While government support measures, including the JobKeeper and JobSeeker payments, appear to be assisting vulnerable Australian households, significant financial distress will follow once they are removed or reduced. As loss of income was a major factor in our analyses, effective government responses should center around providing opportunities for secure employment that pays a living wage, rather than a minimum wage. Additionally, strengthening social protection mechanisms and emergency food relief programs may protect those at risk of food insecurity. Lastly, systems that support the physical access to food, and protect the stability of the food supply must be strengthened. This should involve shortening and localizing food supply chains and bolstering local food systems, where food is grown, packaged, and consumed within the same community. Food policy coalitions could provide the mechanism to work at the intersection of health, social justice and environmental sustainability to improve local and regional food systems, positively influencing the food environment [48,49]. Given the urgent and widely accepted need to transition to a more circular, just, and sustainable economy, the government in partnership with the community should explore setting up appropriately constituted local food councils in each region. These could support community food hubs to enhance market access for farmers, create jobs, build resilience, promote local and sustainable food procurement, and ultimately improve food security by increasing access to local, healthy food [50,51].

## Figures and Tables

**Table 1:** Food security proportions by socio-demographic characteristics and impact of COVID-19 on income using Chi-square comparisons.

Demographics	Category	Food Security Status *n* (%)					χ^2^	*p*-Value
		Total	High	Marginal	Low	Very Low		
Age	18–25	28 (2.6)	16 (57.1)	2 (7.1)	8 (28.6)	2 (7.1)	43.7	<0.001
	26–35	117 (11.0)	80 (68.4)	13 (11.1)	12 (10.3)	12 (10.3		
	36–45	201 (18.8)	146 (72.6)	21 (10.5)	24 (11.9)	10 (5.0)		
	46–55	234 (21.9)	174 (74. 4)	29 (12.4)	21 (9.0)	10 (4.3)		
	56–65	284 (26.6)	221 (77.8)	33 (11.6)	28 (9.9)	2 (0.7)		
	65+	157 (19.8)	157 (77.3)	30 (14.8)	13 (6.4)	3 (1.5)		
Gender	Female	840 (76.7)	626 (74.5)	92 (11.0)	86 (10.2)	36 (4.3)	12.2	0.058
	Male	249 (22.7)	185 (74.3)	41 (16.5)	20 (8.0)	3 (1.2)		
	Other	6 (0.6)	5 (83.3)	0 (0.0)	1 (16.7)	0 (0.0)		
Aboriginal and/or Torres Strait Islander	Yes	25 (2.3)	11 (44.0)	2 (8.0)	7 (28.0)	5 (20.0)	31.7	<0.001
	No	1093 (97.7)	804 (73.6)	131 (12.0)	99 (9.1)	34 (3.2)		
Disability	Yes	238 (21.8)	146 (61.3)	36 (15.1)	32 (13.5)	24 (10.1)	49.9	0.002
	No	856 (78.2)	670 (78.3)	96 (11.2)	75 (8.8)	15 (1.8)		
Rurality	Urban	792 (72.2)	612 (77.3)	82 (10.4)	69 (8.7)	29 (3.7)	13.4	0.004
	Rural	305 (27.8)	206 (67.5)	51 (16.7)	38 (12.5)	10 (3.3)		
Region by LGA	South	653 (59.5)	501 (76.7)	69 (10.6)	65 (10.0)	18 (2.8)	9.4	0.152
	North	254 (23.2)	182 (71.7)	39 (15.4)	20 (7.9)	13 (5.1)		
	NW and West	190 (17.3)	135 (71.1)	25 (13.2)	22 (11.6)	8 (4.2)		
Education	University	737 (67.4)	591 (80.2)	78 (10.6)	52 (7.10	16 (2.2)	43.6	<0.001
	Diploma/TAFE	210 (19.2)	135 (64.3)	30 (14.3)	32 (15.2)	13 (6.2)		
	High School	147 (13.4)	90 (61.2)	24 (16.3)	23 (15.7)	10 (6.8)		
Residency	Born in Australia	869 (79.3)	645 (74.2)	109 (12.5)	83 (9.6)	32 (3.7)	25.8	0.002
	Born overseas, citizen	179 (16.3)	144 (80.5)	17 (9.5)	17 (9.5)	1 (0.6)		
	Permanent resident	31 (2.8)	21 (67.7)	4 (12.9)	3 (9.7)	3 (9.7)		
	Temporary resident	17 (1.6)	7 (41.2)	3 (17.7)	4 (23.5)	3 (17.7)		
Relationship status	Married	572 (52.6)	447 (78.2)	69 (12.1)	49 (8.6)	7 (1.2)	48.3	<0.001
	De facto	178 (16.4)	138 (77.5)	17 (9.6)	16 (9.0)	7 (3.9)		
	Never married	140 (12.9)	92 (65.7)	17 (12.1)	19 (13.6)	12 (8.6)		
	Widowed	43 (4.0)	28 (65.1)	9 (20.9)	4 (9.3)	2 (4.7)		
	Divorced	90 (8.3)	62 (68.9)	16 (17.8)	8 (8.9)	4 (4.4)		
	Separated	33 (3.0)	20 (60.6)	4 (12.1)	4 (12.1)	5 (15.2)		
	Partnered, living apart	31 (2.9)	25 (80.7)	1 (3.2)	4 (12.9)	1 (3.2)		
Household status	Couple, no dependents	471 (43.1)	372 (79.0)	56 (11.9)	36 (7.6)	7 (1.5)	58.7	<0.001
	Couple, dependents	307 (28.1)	226 (73.6)	36 (11.7)	35 (11.4)	10 (3.3)		
	Single parent	65 (6.0)	35 (53.9)	9 (13.9)	9 (13.9)	12 (18.5)		
	Living alone	199 (18.2)	145 (72.9)	28 (14.1)	19 (9.6)	7 (3.5)		
	Other (group/share)	51 (4.7)	37 (72.6)	4 (7.8)	7 (13.7)	3(5.9)		
Main shopper	Yes	893 (81.6)	664 (74.4)	107 (12.0)	91 (10.2)	31 (3.5)	5.9	0.434
	No	202 (18.5)	153 (75.7)	25 (12.4)	16 (7.9)	8 (4.0)		
Household income	<$A20,000	76 (7.0)	38 (50.0)	10 (13.2)	16 (21.1)	12 (15.8)	108.8	<0.001
	$20,000–$40,000	153 (14.0)	93 (60.8)	31 (20.3)	17 (11.1)	12 (7.8)		
	$40,000–$60,000	122 (11.2)	88 (72.1)	20 (16.4)	12 (9.8)	2 (1.6)		
	$60,000–$80,000	127 (11.7)	95 (74.8)	11 (8.7)	16 (12.6)	5 (3.9)		
	$80,000–$100,000	137 (12.6)	109 (80.0)	14 (10.2)	13 (9.5)	1 (0.7)		
	$100,000–$160,000	182 (16.7)	151 (83.0)	20 (11.0)	10 (5.9)	1 (0.6)		
	>$150,000	152 (13.9)	136 (89.5)	10 (6.6)	6 (4.0)	0 (0.0)		
COVID-related Job change	Yes	330 (34.6)	217 (65.8)	42 (12.7)	56 (17.0)	15 (4.6)	25.8	<0.001
	No	625 (65.5)	482 (77.1)	81 (13.0)	44 (7.0)	18 (2.9)		
COVID-related Income loss	No loss	720 (65.8)	568 (78.9)	88 (12.2)	47 (6.5)	17 (2.4)	88.5	<0.001
	>25%	172 (15.7)	129 (75.0)	18 (10.5)	21 (12.2)	4 (2.3)		
	25–49%	79 (7.2)	46 (58.2)	9 (11.4)	14 (17.7)	10 (12.7)		
	50–74%	36 (3.3)	19 (52.8)	3 (8.3)	11 (30.6)	3 (8.3)		
	75–99%	17 (1.6)	6 (35.3)	5 (29.4)	5 (29.4)	1 (5.9)		
	100%	18 (1.7)	9 (50.0)	3 (16.7)	3 (16.7)	3 (16.7)		
Government support payments	No, employed	893 (83.9)	682 (76.4)	102 (11.4)	82 (9.2)	27 (3.0)	30.4	<0.001
	Yes, JobKeeper	96 (9.0)	70 (72.9)	14 (14.6)	10 (10.4)	2 (2.1)		
	Yes, JobSeeker	52 (4.9)	25 (48.1)	13 (25.0)	8 (15.4)	6 (11.5)		
	Unemployed no support payments	23 (2.2)	16 (69.6)	1 (4.4)	4 (17.4)	2 (8.7)		
All participants		*N* = 1067	794 (74.41)	128 (12.0)	106 (9.9)	39 (3.7)		

LGA—Local Government Areas. TAFE—Technical and Further Education.

**Table 2:** Distribution of responses to the six-item food insecurity screen across food security status.

HFSSM Question	Response Option	Food Security Status *n* (%)			
		High (*n* = 863)	Marginal (*n* = 144)	Low (*n* = 118)	Very Low (*n* = 43)
The food that (I/we) bought just didn’t last, and (I/we) didn’t have money to get more. In the last 30 days was this:	Often True	0 (0.0)	0 (0.0)	6 (5.1)	12 (27.9)
	Sometime true	0 (0.0)	108 (75.0)	106 (89.8)	29 (67.5)
	Never True	860 (99.7)	35 (24.3)	6 (5.1)	2 (4.7)
	Don’t know or refused	3 (0.3)	1 (0.7)	0 (0.0)	0 (0.0)
(I/we) couldn’t afford to eat balanced meals. In the last 30 days was this:	Often True	0 (0.0)	8 (5.6)	3 (2.6)	21 (44.8)
	Sometime true	0 (0.0)	28 (19.6)	107 (91.5)	21(44.8)
	Never True	862 (99.9)	106 (74.1)	7 (6.0)	1 (2.3)
	Don’t know or refused	1 (0.1)	1 (0.7)	0 (0.0)	0 (0.0)
In the last 30 days did you or other adults in your household ever cut the size of your meals, or skip meals because there wasn’t enough money for food?	Yes	0 (0.0)	0 (0.0)	15 (12.7)	42 (97.7)
	No	859 (99.7)	142 (98.6)	95 (80.5)	1 (2.3)
	Don’t know	3 (0.3)	2 (1.4)	8 (6.8)	0 (0.0)
In the last 30 days, how many days did this happen?	0–2	6 (0.7)	3 (2.1)	5 (4.2)	3 (7.0)
	3+	0 (0.0)	0 (0.0)	10 (8.4)	39 (90.7)
	Missing/skipped	857 (99.3)	141 (97.9)	103 (87.2)	1 (2.3)
In the last 30 days, did you ever eat less than you wanted to because there wasn’t enough money for food?	Yes	0 (0.0)	0 (0.0)	29 (25.0)	41 (95.3)
	No	860 (99.9)	142 (98.6)	84 (72.4)	1 (2.3)
	Don’t know	1 (0.1)	2 (1.4)	3 (2.6)	1 (2.3)
In the last 30 days, were you ever hungry but didn’t eat because there wasn’t enough money for food?	Yes	0 (0.0)	0 (0.0)	12 (10.2)	32 (74.4)
	No	863 (100)	144 (100)	105 (89.0)	10 (23.3)
	Don’t know	0 (0.0)	0 (0.0)	1 (0.8)	1 (2.3)

HFSSM—Household Food Security Survey Module.

**Table 3:** Association between risk factors and food insecurity—univariate and multivariate logistic regression.

Parameter	Level	Univariate				Multivariate			
		OR	SE	95% CI	*p*	AOR	SE	95% CI	*p*-Value
**Demographics**									
Age	/10 years	0.927	0.0268	(0.876, 0.981)	0.009	0.842	0.050	(0.750, 0.945)	0.004
Gender	Female	Reference Category	-	-	-				
	Male	1.012	0.167	(0.732, 1.400)	0.943				
Aboriginal and/or Torres Strait Islander	No	Reference Category	-	-	-	-	-	-	-
	Yes	3.876	1.586	(1.,738, 8.64)	0.001	2.422	1.237	(0.890, 6.589)	0.083
Disability	No	Reference Category	-	-	-	-	-	-	-
	Yes	2.270	0.356	(1.669. 3.087)	<0.001	1.745	0.347	(1.182, 2.577)	0.005
Rurality	Urban	Reference Category	-	-	-	-	-	-	-
	Rural	1.634	0.243	(1.221, 2.187)	0.001	1.824	0.337	(1.280, 2.619)	0.001
Region	South	Reference Category	-	-	-	-	-	-	-
	North	1.304	0.218	(0.940, 1.801)	0.112	-	-	-	-
	North-West and West	1.343	0.248	(0.935, 1.929)	0.111	-	-	-	-
Education	University	Reference Category	-	-	-	-	-	-	-
	Diploma/TAFE	2.249	0.385	(1.608, 3.145)	<0.001	1.787	0.373	(1.187, 2.690)	0.005
	High School	2.564	0.494	(1.757, 3.741)	<0.001	1.632	0.407	(1.003, 2.656)	0.049
Citizenship status	Born in Australia	Reference Category	-	-	-	-	-	-	-
	Born overseas, Citizen	0.700	0.143	(0.469, 1.043)	0.080	0.787	0.194	(0.486, 1.277)	0.333
	Permanent Resident	1.371	0.537	(0.636, 2.956)	0.421	1.160	0.575	(0.438, 3.067)	0.764
	Temporary resident	4.114	2.052	(1.547, 10.94)	0.005	3.501	2.558	(0.836, 14.66)	0.086
Relationship status	Married/De facto	Reference Category	-	-	-	-	-	-	-
	Never married	1.850	0.368	(1.253, 2.730)	0.002	1.453	0.578	(0.665, 3.171)	0.349
	Previously married	1.805	0.336	(1.253, 2.601)	0.002	2.129	0.855	(0.969, 4.677)	0.060
	Living apart	0.851	0.394	(0.343, 2.109)	0.727	0.734	0.524	(0.181, 2.971)	0.665
Household structure	Couple no dependents	Reference Category	-	-	-	-	-	-	-
	Couple w/dependents	1.347	0.232	(0.961, 1.886)	0.083	1.841	0.409	(1.191, 2.846)	0.006
	Single parent	3.221	0.880	(1.885, 5.503)	<0.001	1.173	0.559	(0.462, 2.983	0.737
	Single person	1.399	0.274	(0.954, 2.053)	0.086	0.746	0.305	(0.336, 1.661)	0.474
	Group/share	1.422	0.474	(0.739, 2.734)	0.291	0.664	0.350	(0.237, 1.860)	0.436
Main Shopper	Yes	Reference Category	-	-	-	-	-	-	-
	No	0.929	0.168	(0.651, 1.324)	0.683	-	-	-	-
Household Income in AUD	<$40,000	2.074	0.406	(1.312, 3.046)	<0.001	2.069	0.483	(1.309, 3.271)	0.002
	$40–$80,000	Reference Category	-	-	-	-	-	-	-
	>$80,000	0.525	0.100	(0.272, 0.478)	0.001	0.567	0.128	(0.363, 0.883)	0.012
**COVID Income Variables**									
COVID-related Job change	No	Reference Category				-	-	-	-
	Yes	1.755	0.253	(1.308, 2.355)	<0.001	-	-	-	-
Government support payments	No, employed	Reference Category	-	-	-	-	-	-	-
	Yes, JobKeeper	1.201	0.291	(0.746, 1.932)	0.452	-	-	-	-
	Yes, JobSeeker	3.491	1.007	(1.983, 6.145)	<0.001	-	-	-	-
	No, unemployed no support payments	1.414	0.650	(0.574, 3.483)	0.451	-	-	-	-
COVID-related Income loss	No decrease	Reference Category	-	-	-	-	-	-	-
	<25%	1.246	0.247	(0.844, 1.838)	0.268	1.299	0.305	(0.820, 2.059)	0.265
	25–49%	2.681	0.659	(1.656. 4.339)	<0.001	2.024	0.623	(1.106, 3.703)	0.022
	50–74%	3.343	1.157	(1.697, 6.589)	<0.001	4.056	1.714	(1.772, 9.284)	0.001
	75–99%	6.851	3.533	(2.493, 18.82)	<0.001	7.143	4.541	(2.055, 24.83)	0.002
	100%	3.739	1.794	(1.459, 9.577)	0.006	1.676	0.920	(0.572, 4.914)	0.346

OR: Odds Ratio; SE: Standard Error; 95% CI: Confidence Interval; AOR: adjusted odds ratio. AUD—Australian Dollar.

**Table 4:** Correlation matrix between socio-demographic variables and Food insecurity variables.

Level	Parameter	A	B	C	D	E	F	G	H	I	J	K	L	M	N	O
A	Food Security	1.000														
B	Gender	−0.002	1.000													
C	Aboriginality	−0.107	0.041	1.000												
D	Age	−0.077	0.042	0.040	1.000											
E	Disability	−0.160	0.012	0.038	−0.160	1.000										
F	Region	0.057	0.012	−0.067	−0.009	−0.012	1.000									
G	Rurality	0.100	−0.040	−0.055	0.083	−0.092	0.245	1.000								
H	Education	0.178	0.041	−0.063	0.118	−0.138	0.168	0.124	1.000							
I	Relationship	0.087	0.000	0.007	0.089	−0.081	−0.003	−0.072	0.048	1.000						
J	Household income	0.073	0.002	0.002	−0.083	−0.025	−0.027	−0.116	0.007	0.687	1.000					
K	Main shopper	−0.012	0.278	0.026	0.011	−0.014	0.024	0.003	−0.031	−0.154	−0.181	1.000				
L	Income	−0.151	−0.008	0.001	−0.265	0.206	−0.039	−0.099	−0.177	−0.250	−0.187	0.073	1.000			
M	Residency	0.038	0.019	0.049	−0.044	−0.013	−0.053	−0.061	−0.052	−0.014	0.051	0.048	−0.031	1.000		
N	COVID job change	−0.122	0.063	0.039	0.151	−0.021	0.019	0.023	0.014	0.000	0.003	0.039	−0.001	0.017	1.000	
O	Government support payments	0.109	−0.022	0.032	−0.068	−0.063	0.069	0.034	0.154	0.002	0.016	−0.014	−0.112	−0.005	−0.290	1.000
P	Drop in income	0.137	−0.028	0.015	−0.055	−0.034	0.037	0.062	0.046	−0.035	−0.001	0.029	0.056	0.090	−0.271	0.284

## References

[1] The Food and Agriculture Organization FAO (2002). The State of Food Insecurity in the World 2001.

[2] Jones A.D., Ngure F.M., Pelto G., Young S.L. (2013). What are we assessing when we measure food security? A compendium and review of current metrics. Adv. Nutr..

[3] Hendriks S.L. (2015). The food security continuum: A novel tool for understanding food insecurity as a range of experiences. Food Secur..

[4] Loopstra R., Tarasuk V. (2013). What does increasing severity of food insecurity indicate for food insecure families? Relationships between severity of food insecurity and indicators of material hardship and constrained food purchasing. J. Hunger Environ. Nutr..

[5] Vozoris N.T., Tarasuk V.S. (2003). Household food insufficiency is associated with poorer health. J. Nutr..

[6] Clay E. (2002). Food Security: Concepts and Measurement.

[7] Australian Bureau of Statistics ABS (2015). 4364.0.55.009—Australian Health Survey: Nutrition—State and Territory Results, 2011-12.

[8] Temple J.B. (2008). Severe and Moderate Forms of Food Insecurity in Australia: Are They Distinguishable?. Aust. J. Soc. Issues.

[9] McKay F.H., Haines B.C., Dunn M. (2019). Measuring and understanding food insecurity in Australia: A systematic review. Int. J. Environ. Res. Public Health.

[10] Kleve S., Davidson Z.E., Gearon E., Booth S., Palermo C. (2017). Are low-to-middle-income households experiencing food insecurity in Victoria, Australia? An examination of the Victorian Population Health Survey, 2006–2009. Aust. J. Prim. Health.

[11] Temple J.B. (2018). The association between stressful events and food insecurity: Cross-sectional evidence from Australia. Int. J. Environ. Res. Public Health.

[12] Foley W., Ward P., Carter P., Coveney J., Tsourtos G., Taylor A. (2010). An ecological analysis of factors associated with food insecurity in South Australia, 2002–7. Public Health Nutr..

[13] Nord M., Coleman-Jensen A., Gregory C.A. (2014). Prevalence of U.S. Food Insecurity Is Related to Changes in Unemployment, Inflation, and the Price of Food.

[14] Sakzewski E. Panic-Buying, Restrictions and the ‘New Normal’: Documenting Life in Australia during the Coronavirus Pandemic. https://www.abc.net.au/news/2020-06-07/australians-documenting-life-in-iso-coronavirus-national-musuem/12251758.

[15] Australian Bureau of Statistics ABS (2020). 6160.0.55.001—Weekly Payroll Jobs and Wages in Australia, Week ending 27 June 2020.

[16] Thomsen A., O’Neill E., Martin Hobbs B., Solomon L. (2020). COVID-19 and Consumers: From Crisis to Recovery.

[17] Kinsella E. (2020). Online sales halted, supermarket shelves stripped bare as shoppers prepare for coronavirus quarantine. ABC News.

[18] Leo Brown S. (2020). Restaurants and cafes facing mass closures without more coronavirus support, industry group warns. The Money ABC News.

[19] Laraia B. (2013). Food Insecurity and Chronic Disease. Adv. Nutr..

[20] Australian Bureau of Statistics ABS (2016). Tasmania 2016 Census QuickStats.

[21] United States Department of Agriculture USDA Survey Tools. http://www.ers.usda.gov/topics/food-nutrition-assistance/food-security-in-the-us/survey-tools.aspx.

[22] Rose D., Oliveira V.J., U.S. Department of Agriculture, Economic Research Service (1997). Validation of a Self-Reported Measure of Household Food Insufficiency with Nutrient Intake Data.

[23] Bickel G., Mark N., Cristofer P., William H., John C. (2000). Guide to Measuring Household Food Security, Revised March 2000.

[24] Butcher L.M., O’Sullivan T.A., Ryan M.M., Lo J., Devine A. (2019). Utilising a multi-item questionnaire to assess household food security in Australia. Health Promot. J. Aust..

[25] Department of Health Tasmania (2020). Report on the Tasmanian Population Health Survey 2019.

[26] Wolfson J.A., Leung C.W. (2020). Food Insecurity and COVID-19: Disparities in Early Effects for US Adults. Nutrients.

[27] Niles M., Bertmann F., Morgan E., Wentworth T., Biehl E., Neff R. (2020). Food Access and Security during Coronavirus: A Vermont Study.

[28] STATCAN (2020). Food Insecurity during the COVID-19 Pandemic, May 2020.

[29] Loopstra R. (2020). Vulnerability to Food Insecurity since the COVID-19 Lockdown.

[30] Lombe M., Chu Y., Wang K., Nebbitt V.E. (2018). The impact of the recession on food insecurity among households who were low income: Findings from the 2005–2014 national health and nutrition examination surveys. J. Poverty.

[31] Australian Council of Social Service ACOSS (2020). Survey of 955 People Receiving The New Rate of JobSeeker and Other Allowances.

[32] Coleman-Jensen A., Gregory C., Singh A. (2014). Household food security in the United States in 2013. USDA-ERS Econ. Res. Rep..

[33] Leroux J., Morrison K., Rosenberg M. (2018). Prevalence and Predictors of Food Insecurity among Older People in Canada. Int. J. Environ. Res. Public Health.

[34] Temple J.B. (2006). Food insecurity among older Australians: Prevalence, correlates and well-being. Australas. J. Ageing.

[35] Quine S., Morrell S. (2006). Food insecurity in community-dwelling older Australians. Public Health Nutr..

[36] Jung N.M., de Bairros F.S., Pattussi M.P., Pauli S., Neutzling M.B. (2017). Gender differences in the prevalence of household food insecurity: A systematic review and meta-analysis. Public Health Nutr..

[37] Pollard C.M., Nyaradi A., Lester M., Sauer K. (2014). Understanding food security issues in remote Western Australian Indigenous communities. Health Promot. J. Aust..

[38] Ferguson M., Brown C., Georga C., Miles E., Wilson A., Brimblecombe J. (2017). Traditional food availability and consumption in remote Aboriginal communities in the Northern Territory, Australia. Aust. N. Z. J. Public Health.

[39] Markwick A., Ansari Z., Sullivan M., Parsons L., McNeil J. (2014). Inequalities in the social determinants of health of Aboriginal and Torres Strait Islander People: A cross-sectional population-based study in the Australian state of Victoria. Int. J. Equity Health.

[40] Caraher M., Coveney J. (2004). Public health nutrition and food policy. Public Health Nutr..

[41] Altman C.E., Heflin C.M., Patnaik H.A. (2020). Disability, food insecurity by nativity, citizenship, and duration. SSM Popul. Health.

[42] National Rural Health Alliance: NHRA (2016). Food Security and Health in Rural and Remote Australia.

[43] Killmorgen A. Coronavirus and Price Gouging—Let’s Put a Stop to It. https://www.choice.com.au/shopping/online-shopping/selling-online/articles/coronavirus-and-price-gouging.

[44] Borjas G.J. (2004). Food insecurity and public assistance. J. Public Econ..

[45] McKechnie R., Giskes K., Gallegos D., Turrell G. (2018). Single-item measure of food insecurity used in the National Health Survey may underestimate prevalence in Australia. Aust. N. Z. J. Public Health.

[46] Thapa D.K., Visentin D.C., Hunt G.E., Watson R., Cleary M. (2020). Being honest with causal language in writing for publication. J. Adv. Nurs..

[47] Parnell W., Gray A. (2014). Development of a food security measurement tool for New Zealand households. Br. J. Nutr..

[48] Caraher M., Carey R., McConell K., Lawrence M. (2013). Food Policy Development in the Australian State of Victoria: A Case Study of the Food Alliance. Int. Plan. Stud..

[49] McCartan J., Palermo C. (2017). The role of a food policy coalition in influencing a local food environment: An Australian case study. Public Health Nutr..

[50] Gale F., Kerslake F., Lewis G., Murray S. (2020). Tasmanians Seeking More Circular and Sustainable Food Systems.

[51] Rose N. (2017). Community food hubs: An economic and social justice model for regional Australia?. Rural Soc..

